# Exploring Gendered Perspectives on Personality Traits and Entrepreneurial Performance in Lebanon during the COVID-19 Crisis

**DOI:** 10.1007/s12147-025-09350-2

**Published:** 2025-01-27

**Authors:** Moustafa Haj Youssef, Nagham Sayour

**Affiliations:** 1 Liverpool John Moores University, Liverpool Business School Redmonds Building Brownlow Hill, Liverpool, L3 5UG UK; 2 Zayed University, College of Interdisciplinary Studies, Academic Building B, Dubai, L2-026 United Arab Emirates

**Keywords:** Gender, Personality Traits, Entrepreneurship, Lebanon, Crisis, Covid-19

## Abstract

We investigate the impact of gendered personality traits on the entrepreneurial performance of male and female entrepreneurs in Lebanon during the COVID-19 crisis. Using the Big Five personality model and survey data from 500 entrepreneurs, the research examines how traits such as agreeableness, conscientiousness, neuroticism, extraversion, and openness to experience influence business outcomes in high-pressure environments. The findings reveal that agreeableness and neuroticism benefit female entrepreneurs more than males during crises, while conscientiousness significantly deteriorates the financial performance of female entrepreneurs. Extraversion and openness exhibit no differential effects on financial performance by gender. By examining the interplay between gendered personality traits and entrepreneurial performance within the unique context of Lebanon during the COVID-19 pandemic, this study contributes novel insights to the literature. It challenges traditional views on gendered advantages in entrepreneurship, particularly by highlighting the underexplored positive implications of neuroticism and the nuanced role of agreeableness. These findings provide actionable insights for policymakers and support organisations. Specifically, fostering relational skills such as agreeableness and leveraging neuroticism’s sensitivity for proactive crisis management can enhance entrepreneurial resilience. Additionally, training programmes aimed at addressing the rigidity associated with conscientiousness can help entrepreneurs adapt more effectively to volatile environments. By tailoring interventions to gender-specific personality dynamics, these insights can strengthen entrepreneurial ecosystems and improve resilience in times of uncertainty.

## Introduction

Personality traits, particularly those in the Big Five Model—extraversion, agreeableness, conscientiousness, neuroticism, and openness to experience—are central to research in psychology, behavioural science, and social sciences (Borghans et al., 2008; Becker et al., 2012; Engle-Warnick et al., 2020). Gender differences in these traits have been widely studied, with findings often attributed to both biological influences and cultural socialisation (Schmitt et al., 2008). For example, women generally score higher on agreeableness, conscientiousness, and neuroticism, while openness and extraversion reveal more complex patterns (Vedel, 2016; Weisberg et al., 2011). These variations highlight the intricate interplay between gendered personality traits and individual behaviours, including entrepreneurial decision-making. Entrepreneurship is a key domain for studying personality traits, particularly during crises like the COVID-19 pandemic. The pandemic brought extreme uncertainty and economic disruption, requiring entrepreneurs to demonstrate resilience, adaptability, and innovation—qualities closely tied to Big Five dimensions (Obschonka et al., 2020). Crises provide a unique lens for examining how personality traits influence male and female entrepreneurs differently, yet such gendered analyses, especially in underexplored regions like Lebanon, remain scarce.

This study examines how personality traits impact entrepreneurial performance across genders during the COVID-19 pandemic, with Lebanon as the focal context. Lebanon’s socio-economic instability—exacerbated by the pandemic—offers a rich setting for exploring entrepreneurial behaviours under stress. The country’s prolonged economic crises, political turmoil, and entrenched patriarchal norms create a challenging environment for entrepreneurs, particularly women. Despite these obstacles, women’s participation in entrepreneurship has grown, presenting a compelling case for investigating how personality traits interact with gender to influence entrepreneurial outcomes. The findings not only illuminate the Lebanese context but also offer insights applicable to other developing economies with similar socio-economic challenges.

Building on prior research, this study explores the role of the Big Five personality traits—agreeableness, extraversion, conscientiousness, neuroticism, and openness to experience—in shaping entrepreneurial performance during crises. While the immediate focus is the COVID-19 pandemic, the implications extend to other crises, such as economic recessions, natural disasters, or political instability. Traits like agreeableness and conscientiousness are known to significantly influence decision-making and resource mobilisation in high-stress environments, regardless of the crisis type (Obschonka et al., 2020). By situating the study within a broader crisis context, this research contributes to the discourse on gendered entrepreneurship and provides actionable insights for navigating uncertainty. This study aims to achieve two objectives. First, it examines gender differences in personality traits among Lebanese entrepreneurs, addressing a gap in the literature on gendered traits in developing countries. Understanding these differences is essential, as findings from developed countries often fail to account for the distinct challenges faced in developing economies. (Christodoulou et al., 2024). Second, it investigates how gendered personality traits influence entrepreneurial performance during crises, offering novel insights into entrepreneurial resilience and adaptability. These findings have practical implications for entrepreneurs, business support organisations, and policymakers, providing strategies to enhance crisis management in entrepreneurship.

Beyond theoretical contributions, this study offers practical insights into improving entrepreneurial resilience. For example, the positive association between agreeableness and financial performance for female entrepreneurs highlights the importance of fostering collaboration and relational skills. Conscientiousness, while often beneficial, may hinder adaptability in turbulent situations when overexpressed. Neuroticism, often viewed negatively, is shown to enhance responsiveness to market challenges when managed effectively. These findings can inform tailored interventions, such as training programmes to develop flexibility and relational skills, mental health support initiatives, and targeted policies to address gender-specific challenges. By enhancing entrepreneurial ecosystems, particularly in regions like Lebanon, this research aims to foster resilience and adaptability among entrepreneurs in the face of future uncertainty.

## Literature Discussion and Hypotheses Development

Personality traits have long been recognised as key predictors of entrepreneurial success and organisational performance (Allison et al., 2024). The entrepreneurial literature has extensively explored the complex ways in which human behaviour, underpinned by these traits, influences entrepreneurial outcomes. Psychological patterns, shaped by individual experiences and environmental factors, play a crucial role in determining entrepreneurial behaviours (Liu, Botella Carrubi, & Blanco, 2024). While these traits can be inherited or developed, they contribute to entrepreneurial resilience, innovation, and success. However, there is limited understanding of how these traits function during crises.

One of the most widely adopted frameworks in entrepreneurship research is the Big Five Model, which categorises personality into five core dimensions: extraversion, openness to experience, conscientiousness, agreeableness, and neuroticism (Franco & Prata, 2019). This model is particularly valuable in examining entrepreneurial behaviour, as it helps illustrate how personality traits influence decision-making, risk tolerance, and leadership styles (Obschonka et al., 2020). The Big Five Model has been applied in various entrepreneurial contexts, including work-life balance, family business dynamics, self-efficacy, and gender-specific entrepreneurial intentions (Zhao & Jung, 2018; Şahin et al., 2019; Parra et al., 2022). Despite its broad application, it is essential to consider how these traits may manifest differently across genders, especially during crises like the COVID-19 pandemic. Entrepreneurship has historically been viewed as a male-dominated field (Hussein & Haj Youssef, 2023), where traits traditionally seen as “masculine”—such as risk-taking, assertiveness, and dominance—are often considered vital for success. However, as more women engage in entrepreneurship, there is increasing interest in understanding how gendered traits impact entrepreneurial outcomes (Huang & Liu, 2016). Traits typically associated with femininity, such as empathy, sensitivity, and compassion, have often been undervalued in entrepreneurial literature. Yet, these traits can be instrumental in building trust, fostering innovation, and managing uncertainty (Huang & Liu, 2016). The COVID-19 crisis provides a unique opportunity to explore how such traits may enable women entrepreneurs to demonstrate resilience, adaptability, and relational leadership, particularly in Lebanon, where entrepreneurship has become a critical survival mechanism amid economic challenges.

Research suggests that individuals displaying more traditionally masculine traits tend to achieve higher-ranking positions and receive greater financial rewards within entrepreneurial ecosystems, reinforcing gender disparities (Drydakis et al., 2018). However, traits should not be viewed as fixed attributes aligned with biological sex. Both men and women possess a mix of masculine and feminine traits, shaped by their experiences and social contexts. In some cases, traditionally feminine traits have proven advantageous for female entrepreneurs, particularly in cultures where emotional intelligence and relational dynamics are highly valued. Huang and Liu’s (2016) research on Chinese female entrepreneurs is a case in point.

Lebanon’s socio-political landscape, combined with ongoing economic challenges, presents a critical context in which to examine how personality traits and gender intersect within entrepreneurship. Female entrepreneurs in Lebanon face unique hurdles, such as limited access to capital and the instability of the political environment. These circumstances make it even more important to understand how personality traits contribute to entrepreneurial success. Systemic barriers often limit women’s access to entrepreneurial opportunities and lead to wage gaps, even when they demonstrate similar competencies to their male counterparts (Drydakis et al., 2018). This gendered reality highlights the importance of examining the role of personality traits, particularly in times of crisis.

We next consider each trait separately. Agreeableness, which includes traits like cooperation, empathy, and trustworthiness, is crucial for building relationships and promoting teamwork in entrepreneurial contexts (Zhao & Jung, 2018). In times of crisis, such as the COVID-19 pandemic, these traits take on even greater significance as entrepreneurs must rely on their ability to communicate openly, manage uncertainty, and establish supportive networks with stakeholders (Allison et al., 2024). Research indicates that women tend to score higher in agreeableness than men, suggesting that female entrepreneurs may leverage this trait to foster collaboration and build resilient networks, which are essential for navigating challenging circumstances (Sharma & Sharma, 2021).

Empirical studies have shown that agreeableness positively influences various aspects of business performance, particularly in relational contexts (Dant et al., 2013). The collaborative nature of agreeableness may enable female entrepreneurs to maintain stronger relationships with teams, suppliers, and customers during the crisis, thereby enhancing their overall performance. Female entrepreneurs, who often exhibit a greater propensity for empathy and teamwork, can use their agreeableness to cultivate supportive environments that facilitate problem-solving and innovation in response to rapidly changing market conditions (Dimitriadis et al., 2018).

In contrast, while male entrepreneurs traditionally benefit from assertiveness and competitiveness—traits often considered advantageous in high-risk environments—the relational aspects of agreeableness may not enhance their performance to the same extent during crises. The ability to build trust and manage relationships effectively can be more beneficial in times of uncertainty, where the challenges presented in crises times, such as by the COVID-19 pandemic, necessitate cooperation and support (Haj Youssef et al., 2024).

Thus, we propose:

### Hypothesis 1

*Agreeableness will have a more positive impact on the entrepreneurial performance of female entrepreneurs than male entrepreneurs during the COVID-19 crisis.*

Extraversion, characterised by strong social skills, expressiveness, and high levels of interaction, is a key personality trait influencing entrepreneurial success. Individuals with high extraversion tend to be more approachable, energetic, talkative, and sociable—traits essential for building relationships and expanding networks (Zhao & Jung, 2018; Franco & Prata, 2019). In entrepreneurial contexts, extraversion enhances resilience and contributes positively to subjective well-being, both of which are crucial for maintaining motivation and managing stress, particularly during crises (Soni & Bakhru, 2023). Entrepreneurs high in extraversion are adept at forming and maintaining relationships with stakeholders, a critical factor in securing resources and gaining support for new ventures (Laouiti et al., 2022). This trait has also been associated with stronger entrepreneurial intentions, particularly in men, where extraversion drives business initiation and leadership. In contrast, female entrepreneurs tend to rely more on traits like openness, neuroticism, and conscientiousness to shape their entrepreneurial intentions, suggesting that extraversion plays a less central role for them (Laouiti et al., 2022). Interestingly, extraversion is positively correlated with higher wages in jobs requiring non-routine tasks, where social interaction and relationship-building are key (Rohrbach et al., 2023). However, in routine jobs, where attention to detail is critical, highly extraverted individuals may struggle, potentially overlooking essential details that impact business performance. In entrepreneurial roles, which often require a balance between strategic oversight and operational management, extraverted entrepreneurs may excel in leadership and networking but encounter challenges in areas that demand precision and focus. Gender differences in extraversion are well documented. Research suggests that men tend to score higher in assertiveness and excitement-seeking—dimensions of extraversion linked to entrepreneurial leadership (Shchebetenko et al., 2020; Costa et al., 2001). Cultural influences also contribute to this disparity. In more conservative settings, such as Tunisia, social norms encourage men to display higher levels of extraversion than women (Hachana et al., 2018). Conversely, women tend to score higher in warmth and enthusiasm—traits that foster nurturing relationships but may not translate as strongly into the assertive leadership often required in entrepreneurship (Weisberg et al., 2011). During the COVID-19 crisis, extraversion took on heightened significance as entrepreneurs relied on their social networks and adaptability to navigate economic uncertainty (Haj Youssef et al., 2024). However, men’s higher levels of assertiveness within extraversion may have provided them with an advantage in seizing opportunities and taking calculated risks, while women’s relational warmth may have been more beneficial in maintaining customer loyalty and team cohesion. Thus, we propose:

### Hypothesis 2

*Extraversion will have a stronger positive impact on the entrepreneurial performance of male entrepreneurs than female entrepreneurs during the COVID-19 crisis.*

Conscientiousness, characterised by traits such as responsibility, organisation, achievement orientation, honesty, and competence, is essential in both personal and professional contexts. Individuals who score high in conscientiousness are typically reliable, punctual, and diligent, traits that contribute to success in various settings (Franco & Prata, 2019; Javid & Abdullah, 2022). Conscientiousness has been shown to positively impact well-being by reducing burnout and increasing a sense of accomplishment, which ultimately improves work-life balance (Parra et al., 2022). Research has also found that females, especially in academic contexts, tend to be more organised, diligent, and hardworking, strengthening the link between conscientiousness and their well-being (Sharma & Sharma, 2021). In entrepreneurship, conscientiousness is a crucial trait as it drives individuals to be detail-oriented, goal-focused, and persistent. These qualities are vital for leaders who must maintain public support, secure funding, and steer their organisations towards long-term success (Bernardino & Santos, 2016). Entrepreneurs high in conscientiousness are often seen as dependable and competent, which is especially important when seeking external resources and support during crises such as the COVID-19 pandemic. Moreover, conscientiousness is strongly associated with entrepreneurial intention, as it motivates individuals to pursue new ventures and persist in overcoming challenges (Şahin et al., 2019). However, there are potential drawbacks to high conscientiousness. Individuals who are excessively focused on honesty and achievement may struggle in situations that require strategic flexibility or adaptability, such as motivating employees or making difficult leadership decisions. Entrepreneurs who are too rigid in their approach may find their long-term performance hindered, particularly during crises where adaptability and quick decision-making are essential. This is especially relevant in human resource management and team building, where overly rigid leaders may impact team dynamics and limit adaptability. Research consistently shows that women score higher in conscientiousness than men, with this trait often associated with vigilance, punctuality, and other qualities linked to femininity (Vrontis & Thrassou, 2013; Else-Quest et al., 2006; Rahafar et al., 2017). However, this same trait might also limit their ability to be flexible or take risks in situations that require rapid and drastic decision-making, such as in the case of COVID-19. Based on this, we propose:

### Hypothesis 3

*Conscientiousness will have a stronger negative impact on the entrepreneurial performance of female entrepreneurs than male entrepreneurs during the COVID-19 crisis.*

Neuroticism is characterised by traits such as impulsiveness, self-consciousness, and sensitivity to negative emotions, often resulting in higher levels of anxiety and stress (Allison et al., 2024). While it is commonly perceived as a disadvantage in high-pressure environments, particularly within entrepreneurial contexts, neuroticism can also offer unique advantages—especially for female entrepreneurs during crises like the COVID-19 pandemic. Females typically score higher in neuroticism, which may enhance their sensitivity to emotional cues and environmental changes. This heightened awareness allows them to respond effectively to the challenges posed by turbulent economic conditions. Individuals with high neuroticism often exhibit greater attentiveness to potential threats and uncertainties (Costa & McCrae, 1992). This sensitivity can lead to a proactive approach, where neurotic individuals engage in thorough analyses of their surroundings, enabling them to identify potential problems before they escalate. As a result, they may demonstrate a greater capacity for meticulous planning, thereby reducing the likelihood of negative outcomes.

Furthermore, the cautious nature associated with neuroticism prompts these individuals to weigh their options carefully, considering various possible scenarios and outcomes (Suls & Martin, 2005). This cautiousness translates into proactive decision-making, as female entrepreneurs may be motivated by the fear of failure to engage deeply in problem-solving efforts. Research suggests that heightened anxiety can drive constructive behaviours aimed at mitigating risks, such as increased planning, research, and collaboration with others (Davis & Nolen-Hoeksema, 2000).

During times of uncertainty, the relational aspect of neuroticism may further enhance communication and collaboration, allowing female entrepreneurs to build supportive networks essential for navigating crises. The emotional intelligence often accompanying neuroticism can foster stronger relationships with stakeholders, positively influencing business performance in challenging times (Weisberg et al., 2011). In contrast, while male entrepreneurs may also experience the negative aspects of neuroticism, such as stress and indecisiveness, they may not benefit from this trait in the same way as their female counterparts. Therefore, we propose:

### Hypothesis 4

*Neuroticism will have a more positive impact on the entrepreneurial performance of female entrepreneurs than male entrepreneurs during the COVID-19 crisis.*

Openness to experience is characterised by traits such as imagination, creativity, and a willingness to explore new ideas. Individuals who score high on this trait are open-minded, curious, and more inclined to seek out novel experiences and opportunities beyond their usual scope (Javid & Abdullah, 2022). In entrepreneurship, openness to experience plays a crucial role in building and maintaining external networks, as entrepreneurs with this trait are more likely to form relationships outside of their immediate circles, facilitating innovation and competitive advantage (Zhao & Jung, 2018). Openness to experience is also influential in driving entrepreneurial intention, as individuals with high scores in this trait are more willing to embrace new ventures and explore unconventional solutions to business challenges (Şahin et al., 2019). Additionally, openness to experience has been shown to positively affect social entrepreneurial intention (SEI) and social self-efficacy. Research suggests that women who tend to score higher in creativity and imagination, may exhibit stronger SEI than men (Uzzal, Shamsul & Vimolwan, 2024). This indicates that female entrepreneurs may leverage their openness to experience to foster creativity and create social value, especially in resource-scarce environments like Lebanon during the COVID-19 crisis. However, the outcomes of openness to experience can be mixed. While it is associated with exploration and resource mobilisation, excessive exploration may lead to decision-making difficulties and the potential depletion of resources. Entrepreneurs who continuously seek the “next best” alternative may struggle to make definitive choices, hindering growth and overall business performance (Obschonka et al., 2020). In crisis situations, where resources are constrained and rapid decision-making is required, balancing exploration and exploitation becomes critical (Kleindienst et al., 2024). Gender differences in openness to experience are well documented but present mixed evidence. Some studies suggest that men score higher in openness, particularly in intellectual confidence (Marsh et al., 2013; Obschonka et al., 2014), while other research highlights that women tend to be more open to new experiences, especially in recognising aesthetics and emotions (Weisberg et al., 2011). Creativity, a core component of openness to experience, has been found to increase entrepreneurial intention among women, further supporting the idea that female entrepreneurs may benefit from this trait in identifying and implementing innovative solutions during crises (Dimitriadis et al., 2018). During the COVID-19 crisis, openness to experience may have offered distinct advantages to female entrepreneurs by fostering creativity and flexibility in responding to unprecedented challenges. However, the inclination to explore multiple alternatives might have also created decision-making difficulties, negatively affecting business performance. In contrast, male entrepreneurs’ intellectual confidence, associated with openness, may have been more beneficial in swiftly navigating the uncertainties of the crisis. Thus, we propose:

### Hypothesis 5

*Openness to experience will have a stronger positive impact on the entrepreneurial performance of female entrepreneurs than male entrepreneurs during the COVID-19 crisis.*

## Methods

### Sample and Data

Our study investigates the role of gender and personality traits in shaping entrepreneurial innovation during crises. The sample includes 500 established entrepreneurs from Lebanon, with 242 males and 258 females. Lebanon’s entrepreneurial ecosystem is particularly compelling due to its ongoing challenges—financial, political, and health crises—that test the resilience of its entrepreneurs. Studying Lebanon’s entrepreneurial landscape offers insights into how gender and personality traits interact in crisis contexts, providing valuable implications for both policy and practice in similar regions. This is due to the unique challenges faced by entrepreneurs in Lebanon, where political instability, economic collapse, and external shocks create a highly volatile business environment, making entrepreneurship both necessary and extremely challenging. Data collection was part of a larger MENA region research project, employing Computer-Assisted Personal Interviewing (CAPI). The survey was translated into Arabic, ensuring linguistic and cultural accuracy for the Lebanese context. First, CAPI ensured standardisation in survey administration, reducing interviewer variability and enhancing data consistency. By employing a tablet-based system, CAPI minimised the risk of data entry errors and allowed for real-time validation of responses, ensuring accuracy even in a context where trust in formal processes can be low. Additionally, the personal interaction facilitated by CAPI enabled interviewers to address respondents’ questions or concerns directly, fostering greater engagement and reducing potential misunderstandings. This approach was particularly valuable in regions where cultural norms emphasise interpersonal trust in communication. Furthermore, the anonymity provided by the CAPI system reduced social desirability bias, encouraging respondents to provide honest and accurate answers, even on sensitive topics such as financial performance and personality traits. This is particularly important to achieve accurate and meaningful insights in such complex environments (Sun et al., 2021).

The survey used validated scales, including the Big Five personality traits (Donnellan et al., 2006). Specifically, respondents were asked to rate their agreement with specific statements on a scale ranging from 1 to 5, which were then used to construct each personality trait following Donellan et al. (2006). Moreover, entrepreneurs were asked whether COVID-19 affected their income. Based on their responses, we construct a financial performance variable during COVD-19 period, assigning a value of 1 if the entrepreneur’s income remained unaffected by the crisis, and 0 if it was impacted. We use this measure to assess the extent to which gender and personality traits contribute to the financial stability or vulnerability among entrepreneurs during crises. Table 1 reports the descriptive statistics for the whole sample. Among the 500 surveyed entrepreneurs of which 51.6% are women, only 44.2% report that their income was not affected by the COVID-19 pandemic. The matrix of correlation between the different variables used in our analysis is reported in Table 2.

**Table 1:** Descriptive statistics

Variable	(1)	(2)	(3)	(4)
	Mean	Std. Dev.	Min	Max
Financial Performance	0.442	0.497	0	1
Female	0.516	0.500	0	1
Extroversion	24.212	8.014	2	40
Agreeableness	29.29	6.432	10	40
Conscientiousness	30.156	6.322	13	40
Neuroticism	20.144	6.728	3	38
Openness to Experience	25.776	5.689	9	40

Notes: The table reports the descriptive statistics. Columns 1 to 4 report the mean, standard observation, minimum, and maximum, respectively. *N* = 500

**Table 2:** Correlation matrix

	(1)	(2)	(3)	(4)	(5)	(6)	(7)
1. Financial Performance	1						
2. Female	0.064	1					
3. Extroversion	-0.013	0.108	1				
4. Agreeableness	-0.049	0.102	0.461	1			
5. Conscientiousness	-0.030	0.133	0.160	0.400	1		
6. Neuroticism	0.036	-0.210	0.127	0.118	0.048	1	
7. Openness to Experience	0.039	0.073	0.290	0.286	0.458	0.053	1

### Data Analysis

We first examine whether there are significant differences in the Big Five personality traits between male and female entrepreneurs in Lebanon, by comparing whether the means are significantly different across gender, an aspect that is understudied in developing countries in general and the MENA region and Lebanon in particular. Following this, we investigate how gendered personality traits influence entrepreneurial performance during the COVID-19 crisis among Lebanese entrepreneurs. For this purpose, we employ a probit regression model, enabling us to estimate the likelihood that specific personality traits, in interaction with gender, affect entrepreneurs’ financial performance during the pandemic.^1^

Specifically, we run the following regression:

$$\eqalign{{Financial\:Perfomance}_{i} & =\:{\beta\:}_{0}+\:{\beta\:}_{1}{Female}_{i}+\:{\beta\:}_{2}{Extroversion}_{i}+\:{\beta\:}_{3}{Agreeablesness}_{i}\cr & +\:{\beta\:}_{4}{Conscientiousness}_{i}+\:{\beta\:}_{5}{Neuroticism}_{i}\cr & +\:{\beta\:}_{6}{Openness\:to\:Experience}_{i\:}+\:{\beta\:}_{7}{ExtroversionxFemale}_{i}\cr & +\:{\beta\:}_{3}{AgreeablesnessxFemale}_{i}+\:{\beta\:}_{4}{ConscientiousnessxFemale}_{i}\cr & +\:{\beta\:}_{5}{NeuroticismxFemale}_{i}+\:{\beta\:}_{6}{Openness\:to\:ExperiencexFemale}_{i}+\:{u}_{i}}$$

where $\:{Financial\:Perfomance}_{i}$ is a dummy variable that is equal to 1 if individual/entrepreneur *i* responded that his/her income was not affected by the COVID-19 pandemic, 0 otherwise. $\:{Female}_{i}$ is a dummy variable that is equal to 1 if respondent *i* is a women, 0 otherwise. The Big Five personality traits are represented by the variables *Extroversion*, *Agreeableness*, *Conscientiousness*, *Neuroticism*, and *Openness to Experience*. These variables were constructed using the validated scale developed by Donnellan et al. (2006). To measure the gendered effect of personality traits on entrepreneurs’ financial performance during the crisis, we include interaction terms of each of the Big Five personality traits with the gender indicator. Finally, *u*_*i*_ is the error term.

## Discussion of Results

**Fig. 1:**
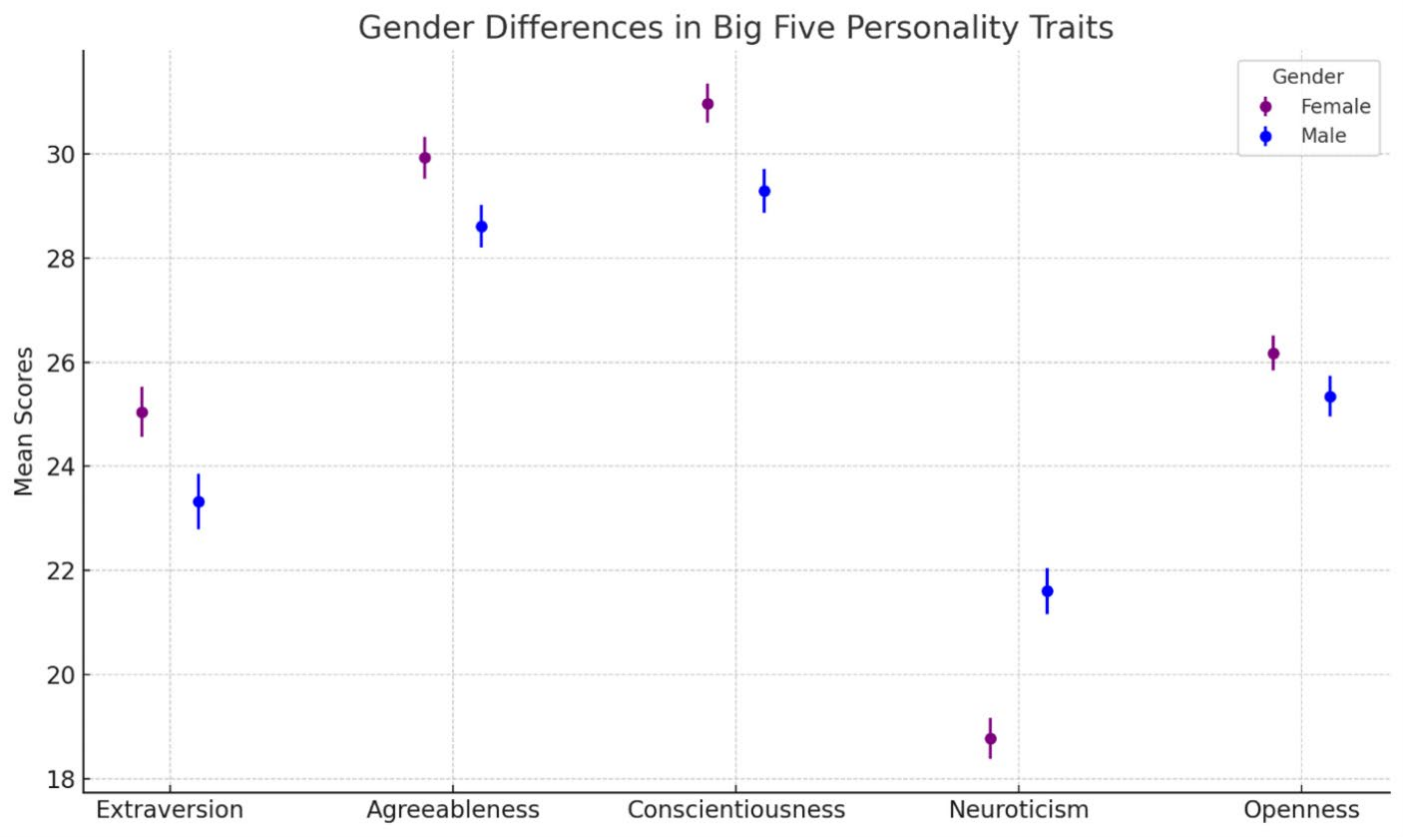
Gender differences in big five personality traits among lebanese entrepreneurs

We first study whether the Big Five personality traits differ significantly among female and male entrepreneurs in Lebanon. Figure 1, plotting the results, shows major gender differences in personality traits. Specifically, we find that females score significantly higher in extraversion than males. This finding is consistent with previous research indicating that women often exhibit higher levels of social engagement and expressiveness, traits associated with extraversion, which can be advantageous in building relationships and expanding networks (Costa et al., 2001; Weisberg et al., 2011). We also find that female entrepreneurs score significantly higher on agreeableness than male entrepreneurs. This aligns with extensive literature that suggests women tend to score higher in traits such as empathy, cooperation, and trust, which are core components of agreeableness (Vedel, 2016; Zhao & Jung, 2018). A similar pattern is found regarding conscientiousness, where Lebanese female entrepreneurs have significantly higher conscientiousness scores than their male counterparts. This is consistent with findings from studies in both Western and non-Western contexts, which indicate that women are generally more detail-oriented, organised, and responsible, traits that are beneficial for entrepreneurial success (Schmitt et al., 2008; Vrontis & Thrassou, 2013). Interestingly, we find a negative and significant difference in neuroticism, implying that Lebanese female entrepreneurs are significantly less neurotic than Lebanese male entrepreneurs. This contrasts with much of the existing literature, which often shows that women typically score higher on neuroticism, reflecting greater sensitivity to stress and anxiety (Costa & McCrae, 1992; Suls & Martin, 2005). However, the unique socio-political and economic challenges in Lebanon may explain this deviation, as female entrepreneurs might have developed greater emotional resilience to cope with ongoing crises, which could have tempered their neurotic tendencies. Lastly, while we find a positive difference in openness to experience, it is not significant, implying that openness to experience is not significantly different between female and male entrepreneurs in Lebanon. This lack of a significant difference is in line with mixed evidence in the literature, where openness tends to vary more by individual characteristics rather than by gender, particularly in entrepreneurial contexts (Weisberg et al., 2011; Marsh et al., 2013).

We then study the impact of gendered personality traits on financial performance during COVID-19. The financial performance is a dummy variable representing financial resilience with the income remaining unaffected during the COVID-19 pandemic. We report the results in Table 3. While we find that women generally fared better than men in terms of financial resilience during the COVID-19 pandemic, this result is not statistically significant. In terms of the Big Five personality traits, we find that agreeableness decreases the probability of being unaffected during a crisis. There is no significant difference in terms of the other personality traits. However, interestingly, we find many significant interaction terms. Specifically, we find that the interaction between agreeableness and female is positive and significant, implying that agreeableness in females may slightly enhance financial performance, counteracting the general negative effect of agreeableness on financial performance; which supports Hypothesis 1. This finding aligns with the argument that female entrepreneurs leverage their higher levels of agreeableness to foster collaboration and build resilient networks, which are crucial in times of crisis (Sharma & Sharma, 2021).We also find support for Hypothesis 3 through the significant negative coefficient on the interaction between conscientiousness and gender. While conscientiousness is often viewed as a positive trait for entrepreneurs, our findings suggest a more complex dynamic, particularly for female entrepreneurs during the crisis. Conscientiousness, typically associated with traits like diligence, responsibility, and organisation, does not independently predict better financial performance. In fact, the negative interaction between conscientiousness and gender indicates that this trait may hinder female entrepreneurs’ performance in a crisis context. This could be due to the tendency for high conscientiousness to manifest as overcaution or perfectionism, which may slow down decision-making and reduce flexibility—key attributes needed in volatile and rapidly changing environments like the COVID-19 pandemic. These findings align with existing literature that recognises conscientiousness as a double-edged sword, particularly in situations that require quick adaptation and risk-taking (Sharma & Sharma, 2021).We also find support for Hypothesis 4 as the interaction of neuroticism with gender has a positive and statistically significant effect. This finding implies that neuroticism may positively influence the financial performance of female entrepreneurs. Although female entrepreneurs in this study score lower on neuroticism compared to their male counterparts, those who do exhibit higher levels of neuroticism may leverage their heightened sensitivity to navigate challenges more effectively. This capability allows them to engage in thorough problem-solving and proactive planning during the crisis. Literature suggests that heightened anxiety can channel neurotic individuals toward constructive behaviours aimed at mitigating risks, facilitating resilience in times of uncertainty (Davis & Nolen-Hoeksema, 2000). Lastly, we observe that the interaction term between extroversion and gender is not significant. A similar result is found for the interaction between openness to experience and gender, implying that both extroversion and openness to experience do not affect the financial performance of female and male entrepreneurs in Lebanon differently. Both results show that there is not enough evidence to support Hypotheses 2 and 5.

**Table 3:** Effects of Gendered personality traits on Financial Performance

VARIABLES	(1)	(2)
	Coefficient	Standard Error
Female	0.026	(0.295)
Extroversion	-0.001	(0.005)
Agreeableness	-0.014**	(0.006)
Conscientiousness	0.004	(0.006)
Neuroticism	-0.001	(0.005)
Openness to Experience	0.009	(0.006)
Extroversion x Female	-0.003	(0.007)
Agreeableness x Female	0.019**	(0.009)
Conscientiousness x Female	-0.018**	(0.009)
Neuroticism x Female	0.013*	(0.007)
Openness to Experience x Female	-0.006	(0.009)
Observations	500	
Pseudo R2	0.029	

Notes: The table reports the marginal effects of a probit model on the effects of gendered personality traits on financial performance. Column (1) reports the marginal effects and column (2) reports the standard errors in parentheses. ***, **, * imply significance at the 1, 5 and 10% level, respectively

**Fig. 2:**
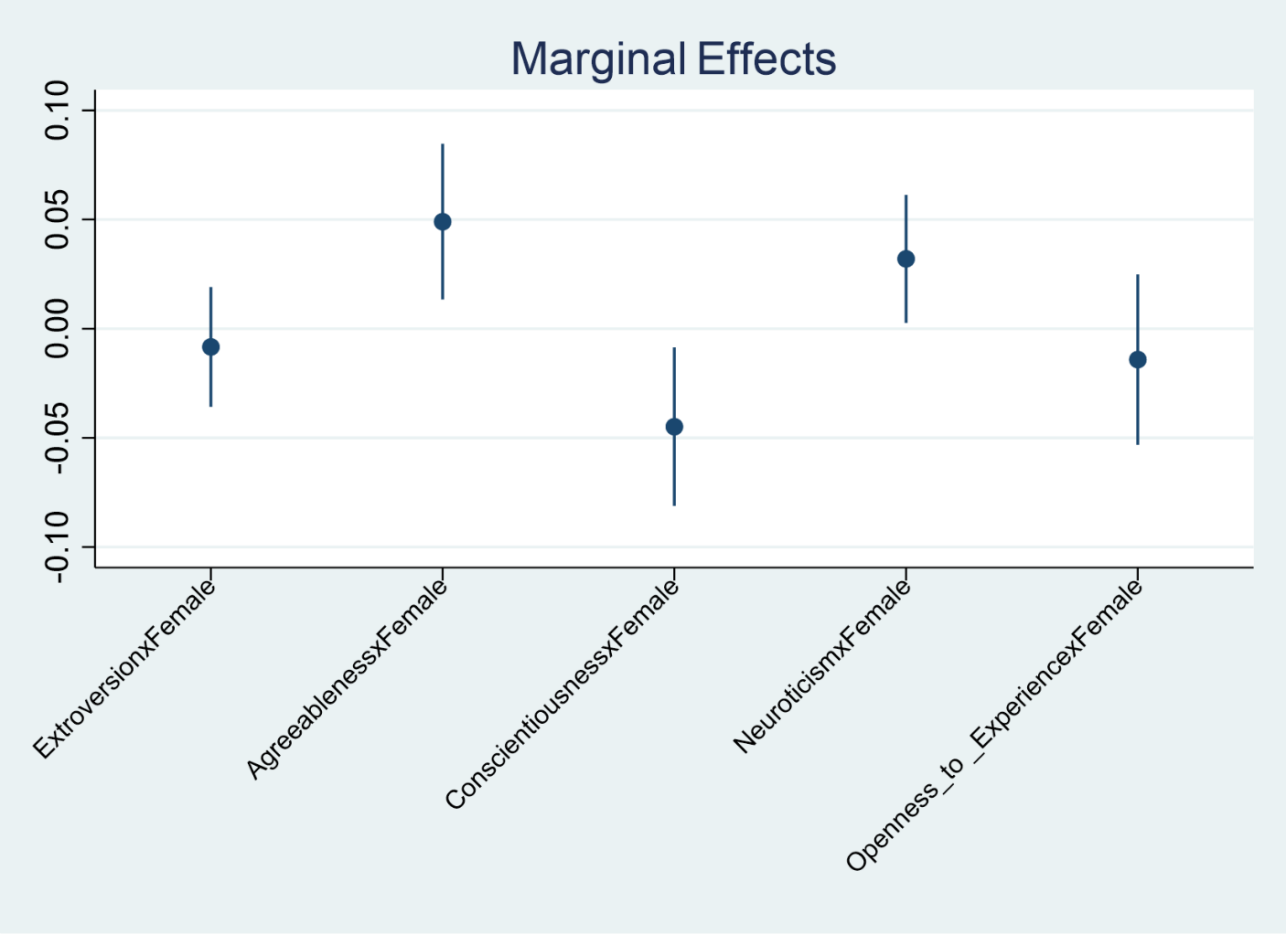
Marginal effects of gendered personality traits on financial performance

To further illustrate the interaction effects between gender and personality traits, we plotted the marginal effects of the interaction terms in Fig. 2. This plot visually demonstrates how the Big Five personality traits affect the probability of financial resilience differently between female and male entrepreneurs. The results reveal that agreeableness has a more pronounced positive impact on female entrepreneurs compared to males, while conscientiousness exhibits a negative impact on female entrepreneurs. Neuroticism, on the other hand, shows a positive effect for females, aligning with our hypothesis that this trait enhances proactive crisis management.

## Implications and Contribution

This study contributes significantly to the literature on gender and entrepreneurship by offering nuanced insights into how the Big Five personality traits interact with gender dynamics in the context of a crisis, specifically during the COVID-19 pandemic. Firstly, it reveals that female entrepreneurs in Lebanon score higher in extraversion, agreeableness, and conscientiousness while being less neurotic than their male counterparts. These findings challenge the traditional notion that entrepreneurial traits are universally applicable across cultures, highlighting the importance of contextual factors in shaping personality traits (Sharma & Sharma, 2021). Secondly, the positive interaction between agreeableness and female entrepreneurs’ financial performance challenges prevailing assumptions that such traits may be detrimental to performance. Instead, it suggests that female entrepreneurs leverage their relational skills to foster collaboration and resilience in times of crisis, which aligns with existing literature that emphasizes the role of social capital and networks in entrepreneurial success (Wasim et al., 2024b; Zhao & Jung, 2018). Furthermore, the study underscores the complex nature of conscientiousness; while it is generally perceived as beneficial, excessive caution may hinder decision-making during turbulent times. In contrast, the positive impact of neuroticism on female entrepreneurs’ performance presents a departure from traditional views that regard neuroticism solely as a liability. This finding suggests that heightened sensitivity may enhance responsiveness to market challenges, ultimately contributing to effective crisis management. Collectively, these contributions enrich the theoretical understanding of gendered personality traits in entrepreneurship, advocating for a more flexible and context-aware approach to studying personality influences on entrepreneurial outcomes.

Our findings have broader implications for understanding how gendered personality traits influence entrepreneurial performance in various crisis scenarios. For example, agreeableness—a trait strongly associated with collaboration and trust-building—may be critical for fostering resilient networks during resource-scarce situations, such as political instability or economic recessions. Prior research highlights that relational skills, inherent in higher agreeableness, are key for navigating uncertainty and building alliances under stress (Zhao & Jung, 2018). Similarly, the positive role of neuroticism for female entrepreneurs, observed in this study, offers insights into crisis management strategies. Neuroticism’s heightened sensitivity to potential risks can foster proactive planning and adaptive decision-making, making it valuable in crises where rapid environmental shifts necessitate careful attention to detail (Davis & Nolen-Hoeksema, 2000). This supports existing literature that reframes neuroticism as an enabler of resilience in high-pressure contexts, provided it is channelled constructively. Conscientiousness, while traditionally beneficial, demonstrated potential drawbacks during crises. The observed negative interaction of conscientiousness with female entrepreneurs in our study suggests that an overemphasis on precision and caution may impede flexibility, a critical component for navigating volatile circumstances. Research has similarly identified the need for balancing conscientiousness with adaptability in crisis situations, particularly where swift, risk-laden decisions are unavoidable (Vedel, 2016). These findings highlight that the interplay of gendered personality traits and entrepreneurial performance is not confined to pandemic contexts but extends to crises that demand resilience, adaptability, and relational competencies. Policymakers and entrepreneurial support organisations can use these insights to develop gender-sensitive training programmes aimed at enhancing traits like agreeableness and neuroticism while mitigating the rigidity of conscientiousness. By doing so, they can equip entrepreneurs with the psychological tools needed to thrive across diverse crisis environments. Policymakers could explore targeted support initiatives designed specifically for female entrepreneurs, leveraging their unique personality strengths. For example, programmes that emphasise relational skill-building, such as mentorship schemes or peer networks, could help female entrepreneurs maximise the positive effects of agreeableness. Similarly, initiatives focused on proactive crisis management, such as access to real-time market intelligence or scenario planning workshops, could enhance the constructive use of neuroticism. Policymakers might also consider offering flexible funding mechanisms that reduce the burden of overly rigid planning associated with conscientiousness, allowing entrepreneurs to pivot and adapt to rapidly changing market conditions.

The findings of this study yield important practical implications for entrepreneurs, educators, and policymakers, particularly in the context of crisis management and support systems. The positive relationship between agreeableness and financial performance for gender-specific entrepreneurs underscores the value of collaboration during crises. This finding suggests that creating environments that promote teamwork, and open communication can enhance resilience. For example, training programmes that develop interpersonal skills and emotional intelligence can equip entrepreneurs with the tools necessary to foster cooperation and empathy. Such programmes might include conflict resolution workshops or networking events that encourage peer support. By building strong, supportive networks, entrepreneurs can better navigate challenges and leverage collective resources, which is critical in volatile situations.

The significant role of conscientiousness in predicting performance highlights the need for structured management practices. Entrepreneurs can benefit from workshops that focus on effective planning, time management, while highlighting the importance of strategic flexibility. For instance, a workshop might introduce project management tools that help entrepreneurs set clear goals while remaining adaptable to changes. By combining diligence with strategic flexibility, entrepreneurs can improve their decision-making processes and operational effectiveness, particularly in unpredictable environments where agility is essential. Educational institutions play a critical role in preparing future entrepreneurs as highlighted by Wasim et al. (2024a, b). Integrating personality assessments into entrepreneurship training programmes can help students identify their strengths and areas for development. For instance, assessments of neuroticism could guide students in learning stress management and emotional regulation techniques, while assessments of conscientiousness might focus on fostering adaptability and strategic flexibility. Additionally, incorporating modules that enhance relational skills, such as communication and networking, could help students leverage agreeableness effectively in collaborative environments. These initiatives would not only develop entrepreneurial competencies but also equip aspiring entrepreneurs with the psychological tools necessary for thriving in diverse economic conditions.

The unexpected positive influence of neuroticism on performance indicates that emotional challenges can also yield constructive outcomes when managed properly. This finding emphasizes the importance of mental health support and stress management resources. For example, establishing support groups or mentorship programmes where entrepreneurs can share their experiences may help them channel their sensitivity into effective problem-solving strategies. Furthermore, integrating mindfulness and stress reduction techniques into entrepreneurial training can help individuals leverage their heightened awareness of challenges to develop proactive solutions. These initiatives not only enhance resilience but also improve overall well-being.

Although this study found no significant impact of openness to experience on performance, fostering a culture of creativity and innovation remains crucial. Encouraging entrepreneurs to engage in creative thinking and risk-taking is particularly vital during recovery phases following crises. Initiatives such as hackathons and innovation challenges can stimulate collaboration and out-of-the-box thinking, enabling entrepreneurs to explore new business models or products. This emphasis on creativity is especially beneficial in rapidly changing markets, where adaptability and innovative solutions drive success. The non-significant effects of openness to experience and extraversion in this study underscore the context-specific nature of personality traits. During crises, immediate survival strategies often outweigh traits associated with long-term growth or social connectivity. Openness, typically linked to creativity and innovation, may hold less relevance in situations demanding pragmatic, swift decision-making (McCrae & Costa, 1997). Similarly, extraversion, characterised by sociability and assertiveness, can be constrained by factors such as social distancing measures or market contractions. These findings suggest that the relevance of these traits may become more pronounced during non-crisis periods, where opportunities for growth and networking are less restricted. Future research should explore this hypothesis through longitudinal studies to assess how the importance of personality traits varies across different phases of economic cycles.

Collectively, these implications contribute not only to the success of individual entrepreneurs but also to the broader entrepreneurial ecosystem in Lebanon and similar contexts. By implementing targeted training and support systems that address the specific needs of entrepreneurs, policymakers can foster a more resilient and dynamic entrepreneurial landscape. This holistic approach encourages collaboration among stakeholders, including government bodies, educational institutions, and private sector organizations, creating a supportive framework that enhances overall entrepreneurial performance.

Despite its contributions, this study raises unanswered questions that warrant further exploration. This study focused on the immediate effects of the COVID-19 crisis, a longitudinal study could provide deeper insights into how personality traits influence entrepreneurial resilience and adaptation over time. Future research could also investigate the intersectionality of personality traits with other factors, such as cultural background, industry type, or socio-economic status, to determine how these variables interact with gender to shape entrepreneurial outcomes. Expanding the geographic scope of research to include diverse regions and economic contexts could offer a more comprehensive understanding of how personality traits influence entrepreneurial performance globally. Furthermore, this study employs financial resilience as the primary measure of entrepreneurial performance, which offers valuable insights into how entrepreneurs navigate crises. While it serves as a meaningful proxy for entrepreneurial performance, it has certain limitations. This measure captures an entrepreneur’s ability to maintain stable income levels despite external shocks, but it does not fully encompass other dimensions of entrepreneurial success, such as innovation, customer retention, or market expansion. Additionally, financial resilience may be influenced by external factors, such as industry-specific dynamics or access to government support, which were not directly controlled for in this study. Future research could benefit from incorporating multi-dimensional measures of entrepreneurial performance to provide a more comprehensive understanding of gendered dynamics.

In conclusion, we highlight the importance of considering both gender and context when examining the impact of personality traits on entrepreneurship. The findings contribute to a deeper theoretical understanding of gender differences in entrepreneurship while offering practical insights for improving entrepreneurial performance during crises. Future research building on these findings will be crucial in advancing both theory and practice in the field of entrepreneurship. By extending the analysis beyond the COVID-19 crisis, this research contributes to a broader understanding of entrepreneurial resilience in diverse contexts. The interplay between gendered personality traits and entrepreneurial performance observed here is likely to hold relevance across various types of crises. These insights not only deepen our theoretical understanding of gender and entrepreneurship but also provide a foundation for developing targeted support mechanisms to enhance entrepreneurial success and resilience during future crises.

## Footnotes

1 As a robustness check, we re-run our regression using an ordinary least square (OLS) methodology. The results are qualitatively similar in terms of sign and significance, and are available from the authors upon request.

## Declarations

### Trial Registration

No clinical trials.
